# Women in clinical trials: a review of policy development and health equity in the Canadian context

**DOI:** 10.1186/s12939-019-0954-x

**Published:** 2019-04-15

**Authors:** Alla Yakerson

**Affiliations:** The Graduate Program in Health, York University, 4700 Keele St, Toronto, ON M3J 1P3 Canada

**Keywords:** Women, Clinical trials, Underrepresentation, Feminism, Sex, Gender, Policy

## Abstract

Health equity in pharmaceutical research is concerned with creating equal opportunities for men and women to partake in clinical trials. Equitable representation is imperative for determining the safety, effectiveness, and tolerance of drugs for all consumers. Historically, women have been excluded from participating in clinical research leading to a lack of knowledge regarding drug effects and their consequences. This paper examines the changes made since the implementation of Canadian policies on the representation of women in clinical trials, the analysis of sex and gender, as well as the discourses that are prominent among researchers. A feminist ethics framework is used to examine the structures that endeavor to elucidate women’s involvement in trials, as experienced under extensive patriarchal history. Scholarly literature and Canadian government policy documents are used to explore the development of clinical trials as pertaining to sex and gender. Findings suggest that women continue to be underrepresented or excluded from important research, highlighting ongoing ethical and justice concerns. Improvement recommendations for policies are outlined.

## Introduction

Health equity is concerned with the attainment of equal opportunities for good health and high quality health care for all people [1]. It requires continuous efforts on behalf of society to address historical and contemporary injustices pertaining to the resources necessary for health including, access to quality healthcare, and safe living and working conditions. When it comes to pharmaceuticals, health equity is concerned with fair rights for all individuals, to the access and participation in clinical research so that the safety, effectiveness, and tolerance of drugs can be determined. This paper seeks to explore the changes made since the implementation of Canadian policies on the representation of women in clinical trials, the analysis of sex and gender, as well as the discourses that are prominent among researchers. In order to do so, this paper will review Health Canada’s policy: *Guidance Document on the Inclusion of Women in Clinical Trials* (1997) and its revision in 2013, the *Health Portfolio Sex and Gender-Based Analysis Policy* (2009) and its revision in 2012, as well as the literature of existing research. A feminist ethics framework will be used to highlight the structures that endeavor to elucidate women’s involvement in trials, as experienced under extensive patriarchal history.

### Biological sex differences

Proper generalizations of drug outcomes to both men and women require researchers to consider the biological differences between the sexes. Pharmacokinetic and pharmacodynamic differences stem from variations in body size, composition, and hormones. Women’s smaller body sizes and higher fat contents typically result in varied drug responses [2]. A study conducted by Ridker et al. [3] showed that aspirin has differential effects on the sexes with regard to primary protection against strokes and heart attacks. As oppose to men, Aspirin only lowered women’s risk of stroke but had no effect on the risk of myocardial infarction or death. In another study, the liver enzyme CY P3A4 showed a slowed drug clearance rate in women when compared to men, resulting in decreased effectiveness of antidepressants, anxiolytics, painkillers and anticonvulsive drugs [4]. In reference to hormonal variations, fluctuating levels of estradiol and progesterone during the female menstrual cycle, drastic increases in hormones during pregnancy, and changes in metabolism as a result of contraceptives, all affect the pharmacokinetics and pharmacodynamics of drugs prescribed [2]. As such, it is evident that in order for women to make informed decisions with respect to their health, drug outcomes must be thoroughly evaluated in both sexes so that comprehensive information about safety, and effectiveness can be made publically available.

### Gender-based differences

In addition to biological variations in sex, gender-based differences must be reflected as they likewise account for variations in drug outcomes. The concept of gender, is broadly used to refer to gender roles, in which women and men are expected to behave in certain ways in society. This social construction of gender norms has important implications for women’s health. Epidemiologically, certain medical conditions are unique to women, while others are more prevalent, reflecting the differences in women’s experiences [5]. For example, women are twice more likely to experience depression at some point in life when compared to men [6]. This has been attributed to the unique experiences of women such as postpartum depression, menopause and the additional stresses of dual work days consisting of paid employment, and unpaid domestic labour (household chores and caregiving for children and elderly). Unfortunately, even when women are included in clinical trials, researchers often fail to determine whether the gender of the study subject affected the outcomes [5].

On the whole, researchers must account for both biological and gender-based differences in their findings in order to properly attribute drug responses. Crucial factors to consider are women’s higher life expectancies leaving them prone to chronic diseases requiring the prolonged use of medications. As such, drugs pertaining to all diseases and conditions experienced by women should be evaluated in clinical trials and generalized appropriately in order to protect and sustain women’s health.

## Historical context

The history of women’s participation in clinical trials is recorded through policies and regulations. “Policy development in the area of protection of human research subjects began in 1949 with the issuance of the Nuremberg Code, which outlined standards for the judgment of flagrantly abusive human experimentation conducted by the Nazis during World War II” ([7]: p2). This document as well as the related declaration of Helsinski (1964) formed the basis for Health Canada and US research regulations. In the mid-1900s, health problems caused by the drugs thalidomide and Diethylstilbestrol (DES), signified abuse and brought about a public awareness for the need for greater protection for fetuses from hazards in medicine. Thalidomide was used by pregnant women in Canada from 1959 to 1962, to prevent morning sickness and was later found to cause peripheral neuritis and severely malformed limbs in newborns [7].

In 1938, DES, a synthetic hormone prescribed to an estimated 200,000 to 400,000 Canadian women to prevent miscarriages, was found 30 years later to have harmful effects on their daughters; reducing fertility and causing adenocarcinoma of the vagina [8]. Following the thalidomide and DES disasters, protective regulations directed towards women of childbearing years became implemented. In the late 1970s, the US Food and Drug Administration (FDA) adopted a policy of exclusion entitled “General Considerations for the Clinical Evaluation of Drugs,” recommending that premenopausal women capable of becoming pregnant be excluded from participating in Phase I and early Phase II of drug trials [9]. Thereby, banning all women from the age of puberty to menopause from pharmaceutical research in order to avoid any likelihood of causing fetal harm. While this policy was supposed to refer only to women of childbearing years and early phases of clinical trials, it quickly translated to all women being excluded from all pharmaceutical research for almost two decades [10].

The lack of information regarding the safety and effectives of pharmaceuticals that were never tested on women led to a strained relationship between physicians and their female patients. Physicians were reluctant to prescribe medications, diagnosed women later or provided them with less aggressive treatments when compared to men [7]. Findings on heart disease demonstrated that women may be diagnosed later than men, and be less likely to be offered invasive procedures such as coronary angioplasty or coronary artery bypass surgery [11]. In addition, lower case survival rates have been observed in women following diagnosis of Acquired Immunodeficiency Syndrome (AIDS), reflecting later identification and less aggressive treatment [11]. Finally, women with kidney disease had difficulty gaining access to dialysis and transplantation, and were later diagnosed with lung cancer and heart disease, resulting in morbidity and mortality [7].

In the 1980s, AIDS activists working to endorse access to experimental therapies presented the first formal challenge to the protectionist policies of the prior decades [11]. They were successful in receiving access to trials utilizing experimental AIDS drugs for the treatment of the serious and life-threatening illness. From then on, women’s advocacy continued with the recognition that clinical outcomes of medications must be evaluated on both sexes.

Concerns over the lack of research on breast and reproductive cancers amplified lobbying for the inclusion of women in clinical trials and in 1997 led to the development of the *Canadian Guidance Document on the Inclusion of Women in Clinical Trials* [10]. The policy served as Health Canada’s promise to develop and apply Gender-Based Analysis (GBA) to programs and policies in order to address the differential needs of men and women. The Guidance Document: *Considerations for Inclusion of Women in Clinical Trials and Analysis of Sex Differences*, was released in 2013 to supersede the 1997 version.

In addition, in the year 2000, Health Canada unveiled another strategy to support a GBA in a document entitled *Health Canada’s Gender-Based Analysis Policy* (GBAP) [8]. The purpose of this reform was to address health differences between men and women as they pertained to sex and gender, understand experiences with health and illness and the interaction with the health care system. Its goals were to be accomplished by “identifying gender equality issues and proposing remedies to inequality in the areas of policy and program development or implementation, research, funding, data collection, surveillance, and regulatory activities” ([8]: p.10). In 2009, the *Health Portfolio Sex and Gender-Based Analysis Policy* replaced the former document. Finally, in 2012, the term “gender-based analysis” was modified to “gender-based analysis plus,” highlighting a new approach which takes into account the intersectional nature of women’s identities and considers factors such as age, education, income, language, geography, and culture [12].

## Methodology/methods

This paper will utilize a feminist ethics theoretical lens to examine the structures that endeavour to elucidate women’s underrepresentation in clinical research, as experienced under extensive patriarchal history. Feminist ethics is an approach to ethics that builds on the notion that traditional ethics have failed to appreciate and value women’s moral experience [13]. Feminists apply a gender-centered approach to ethics in order to rethink and examine issues pertaining to how notions of sex and gender limit and restrict women. By highlighting that society is male-centered, feminists can expose practices of androcentric reasoning in the justification for excluding women from participation in clinical research [14].

Ethical questions concerning policies on the inclusion of women in clinical research seek to determine whose interests are served, and how the policy will affect gender oppression. Feminist ethics scholars are focused on analyzing why women are unjustly excluded or underrepresented and in turn suffer adverse health consequences, how research agendas and decisions are formulated and what discourses continue to promote oppressive practices [15].

Furthermore, the feminist ethics framework appeals to social justice theory that emphasizes the rationales that steer injustices, which privilege some people and harm others through the uneven distribution of power in our society. Social justice seeks to understand processes that underpin injustices and challenge them to promote equity and autonomy [15]. It recognizes that oppression on the basis of gender translates into injustices in the form of marginalization and leads to inequitable health care for women. Thus, when it comes to clinical research policies, feminist ethics and social injustice conceptions investigate how their implementation will affect current practices of power and oppression.

On the whole, by applying a gendered lens, feminist ethics seeks to analyze the norms and assumptions that govern clinical trials in a perspective that allows us to understand how research practices involving and affecting women have undervalued and harmed them. Questions such as whose interests are served and whose are harmed, must be considered in order to highlight the ways in which research has served the interests of privileged social groups and oppressed the marginalized [16]. Thus, in using the feminist ethics framework and appealing to social justice theory we can better recognize the changes that need to be institutionalized if the conduct of clinical research is to meet standards of health equity for women.

Scholarly literature and Canadian government policy documents were used by the author to explore the development of clinical trials as pertaining to sex and gender. Policy documents were taken from Canadian government websites, and scholarly research used to critique the reforms were found through Google Scholar, and databases such as Pubmed.

## Results

### Feminist ethics, social justice and societal norms

Dating back to the third century BC, the great Greek philosopher Aristotle believed that women were imperfect men as they did not reproduce semen and that the female nature should be viewed as afflicted with natural faultiness [17]. Aristotle’s central notion concerning women is that they are by nature mediocre to men and must therefore be subordinate to, and ruled by them. In the early twentieth century, Simone de Beauvoir has had significant influence on feminist ethics by explaining the Hegelian concept of *the Other* which refers to what is unfamiliar and deviating from the norm [18]. Her book *The Second Sex*, views this socially constructed concept as the foundation of women’s oppression in the male-dominated culture. It places the male as standard or the norm in society in relation to women who are the *other* or minority. The process of *othering* is a type of oppression which is endorsed by those who have knowledge, and power and use this to achieve a particular political agenda in its goal of domination [19].

Stemming from this concept of *othering*, Susan Sherwin a contemporary feminist ethics scholar appeals to Iris Young’s social justice theory which identifies oppression as a form of injustice. Young’s notion of social justice recognizes that oppression on the basis of gender translates into injustices in the form of marginalization and leads to unjust health care [15]. Thus, when it comes to clinical trials, feminist ethics and social injustice conceptions of power and oppression, enable a more comprehensive understanding of current research practices which are governed by androcentric reasoning and domination and lead to inequitable health outcomes for women.

### Policies and clinical trials

Guidelines and recommendations for the inclusion of women in clinical trials were developed in response to alarms stressing that harms and injustices were being done to women from consuming drugs for which they have not been tested for [10]. In 1997, the *Guidance Document on the Inclusion of Women in Clinical Trials* was announced by Canadian minister Alan Rock [20]. It recommended that women should be included in all phases of clinical trials in an appropriate sample size that would allow for adequate effects of drug treatments to be evaluated. This in turn, would lead to safer prescriptions and decreased adverse health consequences. Inclusion criteria were directed to all females of childbearing and post-menopausal years, and researchers were encouraged to include women as well as to analyze outcomes of treatments by sex-related differences. In addition, drug manufactures were to safeguard that those drugs seeking market approval included women at all stages of the drug development process to ensure that the full spectrum of risks and benefits were captured throughout the clinical trial. The Committee on the Elimination of Discrimination Against Women, applauded the health ministry with the development of these guidelines stating that it was an important step towards recognizing that women experienced more chronic health conditions, and consumed more medication. Thus, their safety must be acknowledged [21].

In review of the Canadian policy, Canadian guidelines served merely as recommendations for drug manufacturer with no mandatory pre-requisite to conform [10]. The Women’s Health Strategy of Canada [10] promised to monitor the inclusion of women in clinical trials but this was never mandated or put into practice. The Tri Council Policy on the ethical conduct of research involving humans simply stated that, “Women shall not automatically be excluded from research solely on the basis of sex or reproductive capacity,” with no other mention of how this was to be enforced [22].

Guidelines from the National Institutes of Health (NIH) in the US made it a requirement for researchers to include women in clinical trials if they were to be funded [10]. As a follow-up, various investigators including the General Accounting Office in the US assessed the inclusion of women in clinical trials since the implementation of these policies but found their continued underrepresentation in research [10]. Considering that the NIH, having an actual requirement for inclusion failed to bring about change, it is not surprising that in Canada, guidelines that served simply as recommendations, were unsuccessful. In actuality, neither the US or Canada had policies that enforced monitoring provisions for the new guidelines.

In March of 2013, Health Canada released its updated version of the *Guidance Document for Considerations for Inclusion of Women in Clinical Trials and Analysis of Sex* [23]. The document acknowledged that since the 1990s women’s participation in clinical research has increased but underrepresentation continued to be evident especially in the early phases of trials. Furthermore, pregnant and breastfeeding women continued to be excluded leading to gaps in knowledge and safety concerns about the effects of treatments.

The amendments in the 2013 document meant to provide advice to researchers regarding the analysis of sex differences in efficacy of therapies, as well as guidance for inadvertent pregnancy and inclusion of pregnant/breastfeeding women. More specifically, researchers were to carefully plan the design of the trials so that sex differences can be analyzed in a meaningful way, and to provide rationale for any variations in drug responses. Consent forms and counselling were to be offered to all pregnant women regarding potential fetal and reproductive risks. Well understood procedures had to be in place in case inadvertent pregnancy does occur during a clinical trial [23]. Nevertheless, as with the 1997 document, the 2013 guidelines continue to serve merely as recommendations to drug sponsors. To-date, it is still not mandatory to include women and there is no clear indication as to how or if monitoring provisions will ever be enforced.

Literature suggests that while there is no official monitoring for the inclusion of women in research in Canada, it is expected that the enrolment practices are similar to those in the United States [24]. Reports from The Food and Drug Administration (FDA) demonstrate that while women’s participation in clinical trials has progressively improved from < 20% in the 1990s to over 45% between 2010 and 2012, the inclusion in certain cardiovascular and cancer trials remains problematic (24,25). A recent study conducted by Pilote (24) shed light on the underrepresentation of women (24%) in ischemic heart disease (IHD) and heart failure trials, the most common cardiovascular conditions affecting women in the general population. In addition, studies explicitly exclude elderly females from drugs for IHD, even though the disease is more prevalent in older age [25]. Another study looking at the representation of women in Oncology trials from 2003 to 2016, highlighted the continual shortage of female patients’ in trials for melanoma (35%), lung (39%), and pancreatic (40%) cancers [26].

The routine exclusion of pregnant women remains a concern [27]. A recent study concluded that out of 558 Phase IV trials, only five (1%), were designed purposely for pregnant women. The findings also demonstrated that 95% of qualified studies overtly excluded pregnant women, signifying this to be a common practice. As such, this study serves as evidence that industry is not conducting research on pregnant women and highlights the importance of the need for change in practice. In addition, despite the fact that during pregnancy two to three out of five women use four or five medications, there are only a few drugs that are labelled for-use during pregnancy [27]. Data on dosage and safety for most medications on the market remains insufficient. A 2011 study of all therapeutic agents approved by the FDA from 1980 to 2010, found that 91% of these medications did not have sufficient information for their use and risks when taken during pregnancy [28].

In light of these findings, a mandatory shift towards a justification model for excluding pregnant women from important clinical research is recommended. In other words, pregnant women should be included in research unless there is reasonable justification that the tested drug will produce harm to either the mother or the fetus [29].

In review of the GBAP there is no mention that gender and sex-based differences must be analyzed separately for their impact on the experiences of women taking medication. A study conducted at the University of British Columbia’s Centre for Health Services and Policy Research found that GBAP was neither adopted nor implemented in pharmaceutical policy and lacked translation into research practices [30]. Likewise, Doull et al. [31], investigated the use of GBA in 38 Cochrane systematic reviews of cardiovascular health, finding that GBA was largely lacking in the examined literature. Most commonly, if sex or gender were mentioned, the terms were used interchangeably and there was no mention of the intersectional nature of women’s identities [31]. More recently, a study examined the reporting of sex and gender in randomized controlled trials (RCT) across Canada during the period of January 2013–July 2014. The results demonstrated poor analysis of sex and gender, and no analysis of the intersectional nature of women’s identities, in any of the reviewed research [32].

### Discourses among researchers

The Canadian Research Ethics Boards (REBs) examiners reviewed the various assumptions or rationales researchers voice as justification for the underrepresentation or exclusion of women in their clinical trials [33]. Some researchers believe that women are in essence like men in terms of biological similarities and thus finding can be generalized to them. Yet, others acknowledge the biological differences and argue that women introduce too much variation into the data, complicating the results and making interpretation difficult. Thus, it is easier and simpler to study only male participants.

A number of researchers denote that as a result of possible harmful effects from early phases of trials, women are fortunate to avoid participation. However, more often than not, participants do receive favourable outcomes if proper guidelines and safety precautions are followed. They also experience better attendant care and benefit psychologically regardless of being issued the real treatment or a placebo.

As women have traditionally been excluded in trials some researchers inadvertently continue the trend without pondering the consequences or simply state, that women will be included in future research. Difficulties in recruiting and retaining women and expenses that arise due to retention failure, are also rationales for underrepresentation or exclusion. When it comes to enrolling pregnant women, researchers continue to fear harming embryos or fetuses and especially the associated legal liability [33]. It is also a matter of ease as “pregnant women are the only population for which justification for exclusion does not need to be given, making it easy for investigators to avoid issues entirely” ([34]: p.5).

## Discussion

This paper examined the representation of women in clinical trials and the analysis in research by sex and gender*.* Findings from various studies demonstrate that women continue to be underrepresented in clinical research even if conditions such as cardiovascular disease, and certain cancers are more prevalent to them in society. Significant underrepresentation is noted for elderly women and those who are pregnant are often routinely excluded. While the overall representation of women in clinical trials has improved, the analysis of research results by sex, gender and the intersectional nature of women’s identities remains poor.

When the REBs reviewers investigated the causes for underrepresentation and exclusion they found common rationales for the justification of these practices. As such, literature suggests that shared cultural biases continue to dominate medical research and unethical and oppressive practices are still present. In relation to the feminist ethics framework, Simone de Beauvoir’s socially constructed concept of women as the *other* is seen through the continued male standard and dominance of male subjects in clinical trials. Results from research looking at the effects of pharmaceuticals from studies where more men are sampled, are generalized to women with little consideration for differences in sex or gender.

The feminist ethics framework enables us to recognize that andrecentricity continues to demote women’s experiences to a position deviating from the norm and in turn, causes adverse effects to women’s health and wellbeing. According to Susan Sherwin [15], studies often choose to select male subjects, as it is anticipated that women will respond differently and distort the data. Consequently, leaving the practitioner with inadequate knowledge for the treatment of their female patients [35]. In addition, due to the lack of knowledge regarding therapeutic agents, women often choose to avoid taking medications all together. In turn, putting themselves and their fetus at risk for harmful effects.

Appealing to social justice, oppression in society on the basis of gender is still very much present. The passing of the 1997 policy was in theory meant to improve the oppressed status of women from being marginalized to being included in clinical trials. Unfortunately, the uneven sampling of sexes in trials continues to privilege men, highlighting the ongoing unequal distribution of power in our society. As such, it is likely that society’s existing social structures based on male dominance and power have initially stalled the federal government policy from mandating stricter guidelines with enforced provisions that ensure and demand the inclusion of all women in clinical trials. In addition, androcentric structures are the root cause for why researchers themselves have been intuitively less inclined to embrace more inclusive practices.

Upon the release of the 2012, revised draft version of the guidance document: *Draft Guidance Document: Considerations for Inclusion of Women in Clinical Trials and Analysis of Sex Differences* [36], Health Canada sought comments and suggestions for amendments from agencies or the public. The Canadian Women’s Health Network (CWHN), welcomed the opportunity to provide recommendations on the changes [37]. They noted that the document continues to serve simply as a guidance vs. a regulation and thus adherence is understood as being optional. Since Women’s inclusion in clinical trials is imperative, strict monitoring and penalties for non- compliance should be in place. In situations where it is not appropriate to include women in a trial, the document should clearly outline the reasons appropriate for exclusion. Finally, since the terms sex and gender continue to be used interchangeably, there is a need to further educate researchers on how to analyze results accordingly.

The Canadian Alliance for Safe and Effective Use of Medication in Pregnancy, also provided feedback and recommended that the document establish two policies: “one for drugs intended for both male and female consumption and another for drugs intended for women only” ([38]: p.16). In review of the 2013 final version of the document, neither the changes from CWHN nor The Canadian Alliance for Safe and Effective Use of Medication in Pregnancy, were implemented. They continue to be important and should be re-addressed by Health Canada in future revisions.

Recommendations for the GBAP are similar to the guidance policy document. GBA should be mandated in pharmaceutical research with appropriate monitoring. Studies should analyze sex and gender separately for their impact on drug outcomes. Annual reports should be made available to the public in order for both women and men to make informed decisions on the consumption of medication. Finally, researchers should be educated on how to implement the policy in their studies [10].

## Conclusion

There are general guidelines from Health Canada recommending for women to be included in clinical trials as well as a federal government commitment to the application of GBA to research practices. However, at this time these policies are not being sufficiently or properly implemented. Women continue to be underrepresented or excluded from important clinical research resulting in a lack of information regarding vital health outcomes. This lack of clinical data and knowledge regarding prescribed drugs is of concern to feminist ethics. In order to eradicate oppression in clinical trials and step back from *othering* women in research we must first challenge existing social structures of power and dominance by addressing and reframing policies and study agendas. In practice, the inclusion of women in research advances commitment to ethics and justice, and improves the applicability of research findings where the ultimate goal is to ensure equitable health outcomes for both women and men.

## Abbreviations

AIDS: Acquired Immunodeficiency Syndrome
CWHN: Canadian Women’s Health Network
DES: Diethylstilbestrol
FDA: Food and Drug Administration
GBA: Gender-Based Analysis
GBAP: Gender-Based Analysis Policy
IHD: Ischemic Heart Disease
NIH: National Institutes of Health
REBs: Research Ethics Boards
